# “Does a Good Firm Diminish the Bad Behavior of Its Employees?”: The Sequential Mediation Effect of Organizational Trust and Organizational Commitment, and the Moderation Effect of Work Overload

**DOI:** 10.3390/ijerph19116666

**Published:** 2022-05-30

**Authors:** Byung-Jik Kim, Se-Youn Jung, Jeyong Jung

**Affiliations:** 1 College of Business Administration, University of Ulsan, Ulsan 44610, Korea; kimbj8212@ulsan.ac.kr; 2 Department of Psychology, Yonsei University, Seoul 06695, Korea; 3 Prime College, Korea National Open University, Seoul 03087, Korea; 4 Department of Police Science, University of Ulsan, Ulsan 44610, Korea

**Keywords:** corporate social responsibility, work overload, counterproductive work behavior, organizational trust, organizational commitment, moderated mediation model

## Abstract

The purpose of this study was to investigate the influence of corporate social responsibility (CSR) on an employee’s negative behavior, in addition to its intermediating mechanism (i.e., mediators and moderator) in the relationship. This paper proposes that CSR may diminish an employee’s negative behavior, such as counterproductive work behavior. Relying on the context–attitude–behavior framework, this study investigated the mediators and moderator of the relationship between CSR and counterproductive work behavior. Specifically, this study hypothesized that not only does CSR diminish the level of counterproductive work behavior by sequentially boosting the level of employees’ organizational trust and commitment, but their work overload also negatively moderates the association between CSR and organizational trust. Utilizing three-wave time-lagged online survey data from 342 employees in South Korean companies, this study tested the hypotheses by building a moderated mediation model with structural equation modeling analysis. The results indicate that CSR decreases the level of employees’ counterproductive work behavior through enhancing their organizational trust and commitment. Moreover, work overload negatively moderates the association between CSR and organizational trust. The findings of this study make theoretical and practical contributions to the CSR literature.

## 1. Introduction

In recent decades, corporate social responsibility (CSR) has become an increasingly important issue in the management literature, and in business fields [1,2,3]. Although the core concept of CSR has been defined in different ways, it can be described as an organization’s various practices and policies that pursue the enhancement of economic, social, and environmental benefits by fulfilling the needs of the relevant stakeholders (e.g., employees, consumers, suppliers, the local community, governments, the environment) [4,5,6,7,8]. Although there have been various works on the impact of CSR on organizational outcomes, these studies have not yet provided a conclusive answer [2,9,10]. For example, some scholars have reported that CSR functions as an effective strategy to increase the competitive advantage of companies [11,12,13,14]. However, others have found that the relationship is not significant and even negative [7,14]. These inconclusive results call for more research on the relationship between CSR and organizational performance. Against this backdrop, some previous works [2,10,15] have suggested that future studies on CSR need to elaborate the underlying mechanisms (i.e., mediators and moderators) of the CSR–organizational performance link to resolve the lack of solid conclusions.

In an attempt to address this issue, we consider some research gaps in the previous studies on the CSR–organizational outcomes link [2,9,10]. First, previous works on CSR have mainly investigated the influence of CSR activities on macro-level outcomes (e.g., brand value, firm’s reputation, consumer attitudes, financial performance), relatively underexploring the impact of CSR on individual-level outcomes, including employee’s perceptions, attitudes, and behaviors [2,9,10]. Considering that the employee is the main player who substantially strategizes and implements the CSR practices in an organization, an employee’s reactions (i.e., perceptions, attitudes, and behaviors) to the organization’s moral acts (i.e., CSR activities) should be deeply investigated.

Second, many extant works have mainly focused on the positive perceptions or attitudes of employees toward CSR activities, such as organizational commitment, organizational identification, work engagement, and creativity [16,17,18,19,20,21,22], relatively underexploring the importance of employee’s “behaviors” [2,9,10]. In addition, the previous works on CSR have paid less attention to the “negative” aspects of employee’s reactions toward CSR [2,9,10]. Although the positive perceptions and attitudes of employees toward CSR practices are very important in an organization, the perceptions and attitudes may be ultimately expressed in the form of “behaviors” [2,9,10,23]. Thus, this paper suggests that employee’s behaviors are more directly related to a variety of organizational outcomes compared to his or her perceptions or attitudes. In addition, considering that real organizational life is likely to include both positive and negative aspects [2,9], it is critical that an investigation is conducted on the impact of CSR on the negative behaviors, such as deviant behavior and counterproductive work behavior [24,25]. This is the reason why exploring the influences of CSR on employee’s negative behaviors is required.

Third, despite the theoretical and empirical importance, the underlying mechanisms of the CSR-negative behaviors have been relatively underexplored in previous works [2,9,10,24,25]. Through identifying the mediators and moderators, the elaborate explanations about the link can be provided, enabling scholars and practitioners to better understand, predict, and control the CSR–negative behaviors link. This is the reason why examining the association is meaningful.

Fourth, although a number of previous works have investigated a variety of moderating variables with CSR activities (e.g., cultural values, moral values, and personal beliefs about CSR), these studies do not pay sufficient attention to the critical role of job characteristics [9,10]. In other words, to the best of our knowledge, few studies have examined the interaction between CSR activities and employees’ perceptions toward their job characteristics. However, considering that job characteristics are likely to significantly influence an employees’ perceptions, attitudes, and behaviors in an organization [26], examining the moderating effect of job characteristics in the association between CSR activities and a member’s reactions is highly needed.

To address these issues, this study investigated the intermediating mechanisms (mediator and moderator) of the association between CSR and members’ negative behaviors and, specifically, counterproductive work behaviors. Counterproductive work behavior can be defined as a member’s intentional behaviors that directly and indirectly cause harm to the organization’s various stakeholders [27]. Although many scholars have examined the antecedents, mediators, moderators, and outcomes of this behavior [28], few studies have examined the impact of CSR on counterproductive work behavior [24,25].

Specifically, based on the context–attitude–behavior perspective [29], we propose that members’ organizational trust and organizational commitment sequentially mediates the association between CSR and counterproductive work behavior. Previous works on CSR have reported that CSR enhances members’ trust in organizational contexts [9,10]. Organizational trust has been known to function as an important lens that affects member’s perceptions and attitudes when he or she is interpreting a variety of policies, systems, and practices in an organization [29,30,31,32]. This paper proposes that CSR positively affects the level of organizational trust based on the “influential model of trust” theory [33,34]. Then, organizational trust may increase the quality of an employee’s organizational commitment [35,36]. Organizational commitment indicates the extent to which employees not only feel psychological attachment but also intend to contribute to achieving the goals of the organization [37,38,39]. Eventually, employees with a high level of organizational commitment decrease harmful behaviors, such as counterproductive work behavior, that impede the success of the organization [24,25].

Furthermore, this paper proposes that CSR may not always enhance the degree of members’ organizational trust, although the above argument that CSR activities increase the quality of employee’s organizational trust may be reasonable enough. Although there may exist various situational, contextual, or environmental factors, both at the individual and organizational levels, in an organization that may moderate the CSR–organizational trust link, this paper attempts to propose an important individual-level moderator. Among various potential individual-level moderators, this paper focuses on the degree of an employee’s work overload, which has been regarded as one of the most critical job characteristics. Work overload is defined as the degree to which an employee feels that the overall amount or intensity of their work is excessive in an organization [40,41].

Specifically, this paper propose that the degree of work overload may negatively moderate the CSR–organizational trust link. When employees feel a high level of work overload, they think that their company does not fulfill its fundamental responsibility for the internal stakeholders (i.e., employees). Although the firm attempts to achieve good and moral purposes through CSR activities for its external stakeholders, if the employees feel a high level of work overload, they are likely to perceive that the firm does not prioritize the essential needs of the internal stakeholders (e.g., the amount or intensity of work). In this situation, the employees may doubt the real intention of the company, and the authenticity of the CSR activities, feeling that the firm’s CSR has a hidden, impure purpose. This doubt decreases the positive influence of CSR on members’ organizational trust. In other words, the influence of CSR on a members’ organizational trust may depend on the degree of work overload.

In summary, the specific research questions of this paper are as follows:

1. How does CSR affect the negative behaviors of employees (i.e., counterproductive work behavior)?
2. What are the intermediating mechanisms between CSR activities and employees’ counterproductive work behavior?
3. What is the contingent variable that moderates the influence of CSR activities on employees’ attitudes (i.e., organizational trust and organizational commitment)?

To answer these questions, in this paper, we suggest that organizational trust and organizational commitment may function as sequential mediators in the CSR–counterproductive work behavior link. In addition, the degree of employee’s work overload negatively moderates the relationship between CSR and organizational trust.

We believe that this paper may contribute to the CSR and counterproductive work behavior literature. First, this study examined the impact of CSR on an employee’s negative behavior, (i.e., counterproductive work behavior) instead of his or her perceptions or attitudes. Second, this study investigated the sequential mediating role of organizational trust and organizational commitment as an individual-level intermediating mechanism of the link. Third, this study revealed that the degree of work overload, which is one of the critical job characteristics, may negatively moderate the influence of CSR on members’ organizational trust. Fourth, from a methodological perspective, this study attempted to diminish the harmful effect of the common method bias due to a cross-sectional research design that utilized three-wave time-lagged data.

## 2. Theory and Hypotheses

### 2.1. CSR and CWB

Although relatively few works have examined the association between CSR activities and member’s counterproductive work behavior, relying on the extant studies, this paper expects that CSR practices may decrease the degree of members’ counterproductive work behavior [24,25]. Given that the organization’s various activities of caring for and supporting a variety of its stakeholders would be fundamentally moral and ethical [30,31], CSR practices may be considered as being moral and ethical [1,2,3,4,5,6]. When an organization implements its social responsibility well, the employees may think that their organization is moral with a high degree of an ethical climate [30,31]. Then, the employees are likely to accept and follow various ethical practices, activities, and norms that their organization pursues, feeling that they should behave in accordance with this ethical climate. This enhances the quality of an employees’ ethical decisions, in turn boosting their ethical behaviors and diminishing unethical behaviors such as counterproductive work behavior [24,25,30,31]. For instance, Kim and Choi [23] showed that when an organization actively implements its social responsibilities, the degree of an employee’s organizational identification is increased, eventually diminishing the level of his or her counterproductive work behavior. Hur and his colleagues also [25] reported that CSR practices increase the degree of organizational civility norms, which in turn enhance the employees’ job calling, eventually diminishing customer-directed counterproductive work behavior. Thus, this paper expects that CSR may decrease the level of employees’ unethical behaviors [29,30,31,32,33,34,35,36,37,38,39,40,41,42,43,44,45].

### 2.2. CSR and Organizational Trust

Based on the previous theories and studies, this research suggests that CSR may increase the level of employee’s organizational trust [2,9,10,42]. Organizational trust has been known to function as an important lens that affects members’ perceptions and attitudes when he or she is interpreting a variety of policies, systems, and practices in an organization [29,30,31,32]. Therefore, organizational trust can play a crucial role in building a variety of individual members’ outcomes, such as organizational commitment, organizational identification, job satisfaction, and in- and extra-role performance [30,31,32,33,34].

The influence of CSR on organizational trust can be generally explained by the two theories of (1) “social exchange theory” and (2) “influential model of trust” [2,9,10,42]. First, social exchange theoretically supports the association between CSR activities and employees’ organizational trust. The essence of the theory is the principle of reciprocity. In the social exchange context, when one party gives other party some benefits, the receiving party may perceive a strong feeling of obligation that they have to provide the first party with similar things in terms of political, economic, or social value [34,42]. Specifically, employees in an organization that actively implements CSR practices may feel that they work for a socially desirable and admirable organization. This feeling of pride is likely to enhance the quality of their social self, providing a sense of obligation toward the organization in the employees, since their positive self is originated in the organization’s CSR activities. Then, the employees try to repay this sense of obligation by boosting their positive attitudes toward their organization. In other words, CSR practices can increase the level of an employee’s organizational trust by facilitating social exchange processes between employees and their firm [2,9,34,42].

Second, another theoretical explanation can also support the positive influence of CSR activities on an employee’s organizational trust; that is, the “influential model of trust” [33,34]. This perspective suggests that ability, benevolence, and integrity critically determine the level of trust, functioning as an important antecedent of it [33,34]. Ability indicates the knowledge or abilities by which a trustee affects a trustor in a specific field. Benevolence means “the degree to which a trustee is believed to have a will to provide benefits with the trustor, beyond egocentric motive” [33]. Third, integrity indicates the trustor’s perception that the trustee has principles that the trustor feels are acceptable [33,34]. By applying this model in the CSR context, this paper expects that CSR activities increase the degree of organizational trust, since CSR practices can fulfill the criteria of the three antecedents of trust. Specifically, first, when an organization actively implements CSR practices, the members are likely to feel that the organization has enough abilities or resources to be able to afford it. Since they may perceive that firms with sufficient resources can continue to implement CSR activities, the moral practices (i.e., CSR) make them believe that their firm possesses a sufficient level of “ability”. Second, CSR activities can satisfy the criterion of “benevolence”. Considering that employees are both the direct and indirect beneficiaries of CSR activities [9,10,23], they may feel that their organization is benevolent enough to satisfy the second criterion. Lastly, CSR practices make the members think that their organization adheres to moral and social value to maintain integrity. Therefore, CSR activities can fulfill the criterion of “integrity”. Taken together, we suggest that CSR activities can increase the level of employees’ organizational trust being based on the influential model of trust. Relying on the above arguments, this paper proposes the following hypothesis:

**Hypothesis** **1.** *An employee’s organizational trust is positively associated with CSR.*

### 2.3. Organizational Trust and Organizational Commitment

Previous studies have demonstrated that organizational trust is closely associated with employee’s attitudes toward organization, including organizational commitment [29,30,35,36]. Organizational commitment can be defined as the extent to which an employee not only feels psychological attachment but also has the intention to contribute to achieving the goals of the organization [37,38,39]. This concept has been known to be closely associated with positive outcomes, including a high level of job satisfaction, organizational citizenship behavior, and in-role and extra-role performance [38,39].

Organizational trust has been regarded as an important antecedent to boost organizational commitment by enhancing positive relationships among employees [29,30,31,32]. Organizational trust tends to increase the quality of the collaborative atmosphere among members in an organization, supplying them with a sense of psychological safety and attachment toward the organization [29]. By enhancing friendly interactions among members, the feeling of trust builds emotional bonds with his or her organization. Through this process, the employees may feel that they are very valuable and proactive protagonists in the organization. This friendly connection and sense of belonging to their organization creates positive emotions for the members in an organization [32]. Then, these positive emotional reactions are likely to be prevalent among members, who experience a substantial boost in their emotional connection and attachment to their organization in the form of positive attitudes, such as organizational commitment [29,30,31,32]. In short, organizational trust plays the critical role of the social glue to create members’ long-term attachment to their organization.

Furthermore, the association between organizational trust and commitment can also be explained by the social exchange perspective [42]. Based on this theory, this paper suggests that an employee who trusts in his or her organization is likely to repay the positive experiences of the emotional bonds and connection with something positive for the organization. The feeling of a member’s trust toward the organization may make him or her build a good and favorable relationship with his or her leaders and colleagues. The positive experiences that are grounded in organizational trust may enhance a sense of obligation to repay it by increasing positive attitudes toward his or her organization, such as in the form of organizational commitment [35,36]. Based on the above explanations, we suggest this hypothesis:

**Hypothesis** **2.** *An employee’s organizational commitment is positively associated with his or her organizational trust.*

### 2.4. Organizational Commitment and Counterproductive Work Behavior

This paper suggests that member’s organizational commitment diminishes the level of his or her counterproductive work behavior. An employee with a high degree of organizational commitment is likely to perceive a sense of connection with his or her firm, ultimately pursuing the firm’s value systems and goals [37,38,43]. The employee’s feeling of connection enhances his or her positive perceptions and attitudes to it [43], also encouraging him or her to make the best efforts to achieve his or her firm’s goals [44,45]. To achieve the organization’s goals, the members are likely to increase organizational citizenship behavior and cooperative behavior [46,47,48,49,50].

Based on the above arguments, we can expect employees who have a high level of organizational commitment will increase actions that are correspondent with achieving their organization’s goals, and also decrease actions that are not consistent with the direction of the firm’s goals. Thus, an employee with a high level of organizational commitment tends to conduct less deviant behavior that directly harms his or her firm [24,25]. The member may feel that the growth and success of the firm are closely associated with his or her self-concept, directly and ultimately enhancing his or her own growth and success. Then, the member is likely to attempt to diminish harmful behaviors, such as counterproductive work behavior, that impede the success of the organization [24,25]. For example, Kim and Choi [24] found that an employee’s sense of belonging to his or her organization decreases his or her counterproductive work behavior. In addition, Al-Atwi and Bakir [50] reported that a feeling of employee’s oneness toward the organization is likely to diminish his or her counterproductive work behavior toward the organization (CWB-O), in addition to the counterproductive work behavior pertinent to his or her individual-level factors (CWB-I). Relying on the above explanations, this paper proposes the following hypothesis:

**Hypothesis** **3.** *An employee’s counterproductive work behavior is negatively associated with his or her organizational commitment.*

### 2.5. Sequential Mediating Role of Organizational Trust and Organizational Commitment between CSR and Counterproductive Work Behavior

To integrate each of the aforementioned hypotheses in a comprehensive model, this paper proposes that an employee’s organizational trust and organizational commitment sequentially mediate the association between CSR activities and the employee’s counterproductive work behavior. This mediation model theoretically relies on a context–attitude–behavior framework [24,29].

The context–attitude–behavior perspective [29] can be utilized to support this mediation hypothesis. This theoretical framework suggests that there are many social/contextual factors in an organization, including various rules, systems, culture, climate, and practices, which functions as crucial factors to create an employee’s attitudes, eventually building his or her behaviors. CSR practices may play the role of an important social context by forming employee’s attitudes, such as those relating to his or her organizational trust and organizational commitment. Then, the attitudes influenced by this context build his or her behavior, such as counterproductive work behavior.

Relying on these arguments, this paper expects that an employee’s organizational trust and organizational commitment sequentially mediate the association between CSR practices and counterproductive work behavior. Thus, we propose this hypothesis:

**Hypothesis** **4.** *An employee’s organizational trust and organizational commitment sequentially mediate the association between CSR practices and counterproductive work behavior.*

### 2.6. Moderating Effect of Work Overload in the CSR–Organizational Trust Link

Although the hypothesis that CSR enhances the degree of a member’s organizational trust seems reasonable, we believe that the argument may be somewhat naïve since there may exist many situational, contextual, or environmental factors that can directly or indirectly influence the association between CSR and organizational trust in a real organization. In other words, the CSR–organizational trust link may be moderated by the various contextual/situational factors (i.e., moderators). In the current study, we examined the moderating role of job characteristics since the concept has been considered to substantially affect employee’s perceptions and attitudes in an organization [26]. Among a variety of job characteristics, we focused on an employee’s perceived work overload, which has been regarded as one of the most critical job characteristics. Work overload can be defined as the degree to which a member feels that the overall amount or intensity of his or her work is excessive in an organization [40,41]. Previous research has shown that work overload may be one of the most detrimental work-related stressors. According to these studies, work overload tends to decrease the degree of a members’ job satisfaction, organizational commitment, intrinsic motivation, work engagement, identification toward the organization, self-efficacy, and self-esteem. Moreover, the concept is likely to increase the level of a member’s job stress, work–family conflict, burnout, psychological pressure, and intention to leave [51,52,53,54,55,56,57,58,59,60,61,62].

Specifically, this paper proposes that the impact of CSR on the degree of a member’s organizational trust may be moderated by the degree of his or her work overload, given that, when a firm implements social responsibility activities, the employees are the main actors who substantially plan, strategize, and conduct the moral practices. From the employees’ perspective, they should work more and more due to the increased amount and intensity of their work related to CSR activities. In other words, although the firm has good intentions for many stakeholders, its CSR activities are likely to paradoxically increase the physical/psychological burden of employees, who represent one of the most important stakeholders.

For example, when members feel a high degree of work overload, they may perceive that their firm does not satisfy its fundamental responsibility for the internal stakeholders (i.e., employees). Although the company tries to achieve a good and moral purpose through CSR for its external stakeholders, when the employees feel a high degree of work overload, they may think that their firm does not prioritize the essential needs for the internal stakeholders (i.e., work overload). In this situation, the members doubt the real purpose of the firm, in addition to the authenticity of the CSR activities, and feel that the firm’s CSR has hidden impure intention. This doubt diminishes the positive influence of CSR on members’ organizational trust.

**Figure 1:**
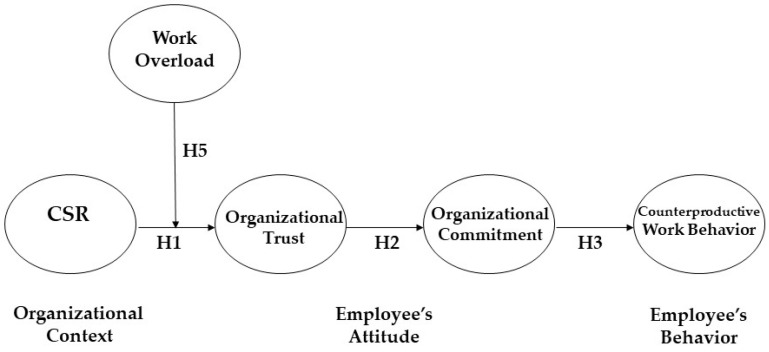
Theoretical model.

In contrast, when members feel a low degree of work overload, they may perceive that their company’s moral activities (i.e., CSR) to help external stakeholders are consistent and authentic. Then, the employees believe that the firm’s moral practices originate in its internal morality, and are more trusting in the authentic intention of the CSR. In this situation, the positive influence of CSR on the members’ organizational trust may be amplified. Based on these arguments, we propose the following hypothesis (Please See Figure 1):

**Hypothesis** **5.** *Work overload negatively moderates the relationship between CSR and organizational trust.*

## 3. Method

### 3.1. Participants and Procedure

To empirically test our hypotheses, in this study we gathered the survey data from employees in South Korean firms through an online survey system. The data collection process was implemented by one of the largest research companies, which possesses the largest population Korean research panelists (approximately 1,800,000). The respondents of this study were randomly selected by the research company; thus, the possibility of biased sampling was reduced. With the online survey system, this study collected data from each respondent at three different time points at intervals of 5~6 weeks, thus diminishing the possibility of the common method bias. By dividing the data collection time points, we reduced the possibility of the bias that originates due to the same respondent measuring entire variables at the same time.

At Time 1, a total of 770 employees participated. Among those participants, 551 employees responded to our survey at Time 2. At Time 3, we received survey data from 357 employees. After eliminating missing data, data from a total of 342 employees were used for analysis. The descriptive features of the respondents are described in Table 1.

### 3.2. Measures

This study asked respondents to evaluate the perceived level of CSR and his or her work overload at Time 1. At Time 2, we gathered data on organizational trust and organizational commitment, and at Time 3, counterproductive work behavior data was collected. All variables of this study were evaluated with multi-item scales on a five-point Likert scale (1 = strongly disagree, 5 = strongly agree). We computed the internal consistency of our research variables through Cronbach alpha values (Please See Appendix A).

#### 3.2.1. CSR (Time Point 1, Collected from Employees)

CSR practices were evaluated by 12 items of Turker’s CSR scale (Turker, 2009). This measure takes the stakeholder approach, which includes items about the social responsibility towards a variety of “stakeholders” such as employees, customers, local communities, suppliers, and the environment. Since it is not practically possible to include all the stakeholders, 4 main actors were selected to represent the overall CSR activities [22]. In the scale, CSR measures for the environment, community, employee, and customer dimensions were selected as the main parts of the overall CSR. To evaluate CSR practices for the environment, 3 items were used, such as “our company participates in activities which aim to protect and improve the quality of the natural environment”. Second, CSR for the community domain also consisted of three items; for example, “our company contributes to campaigns and projects that promote the well-being of the society”. Third, CSR activities for the employee domain consisted of 3 items, including “The management of our company is primarily concerned with employees’ needs and wants”. Lastly, we utilized 3 items on CSR for the customer domain, such as “our company respects consumer rights beyond the legal requirements”. The items were collected from respondents at time point 1. The value of Cronbach’s alpha was 0.91.

#### 3.2.2. Work Overload (Time Point 1, Collected from Employees)

To measure the degree of employee work overload, we used 5 items from previously published scales [63,64]. All items included in our study were: (a) “I am pressured to work long hours”; (b) “I have unachievable deadlines”; (c) “I have to work very fast”; (d) “I have to work very intensively”; and (e) “I have unrealistic time pressures”. The Cronbach alpha value in this study was = 0.91.

#### 3.2.3. Organizational Trust (Time Point 2, Collected from Employees)

At time point 2, three items to measure OT were selected from the scale of Cook and Wall (Cook and Wall, 1980). According to the suggestion of previous research [65], we selected core items of the OT scale. Sample items were “I trust my organization”, and “I feel that my company is reliable”. The value of Cronbach alpha in this study was = 0.90.

#### 3.2.4. Organizational Commitment (Time Point 2, Collected from Employees)

At time point 2, to measure organizational commitment, we utilized 4 items Meyer and Allen’s scale [38]. Sample items were (a) “I really feel as if my organization’s problems are my own”; (b) “I feel a strong sense of belonging to my organization”; (c) “I feel emotionally attached to my organization”. The value of Cronbach alpha of this variable was = 0.93.

#### 3.2.5. Counterproductive Work Behavior (Time Point 3, Collected from Employee’s Immediate Leader)

At time point 3, counterproductive work behavior was measured by utilizing Fox and his colleague’s scale [66], with 5 items. The respondent’s immediate leader evaluated the employee’s degree of counterproductive work behavior. The sample items were “This employee told people outside the job what a lousy place you work for”, “This employee insulted someone about their job performance”, and “This employee purposely worked slowly when things needed to get done.” The value of Cronbach’s alpha was 0.89.

#### 3.2.6. Control Variables

This study controlled for tenure, gender, position, and education of employees since these variables have been known to affect counterproductive work behavior [67,68]. The variables were collected at Time 1.

### 3.3. Analytical Strategy

This study conducted a frequency analysis to check the demographic characteristics of the respondents. Then, the associations among research variables were calculated with Pearson correlation analysis using the SPSS 26 program. Relying on the suggestion of Anderson and Gerbing [69], this paper took a two-step approach, including the measurement model and the structural model. To test the validity of our measurement model, this paper performed a Confirmatory Factor Analysis (CFA). In addition, a moderated mediation model analysis with SEM was conducted to test the structural model with the AMOS 23 program. To test whether the model fit indexes were acceptable, this study utilized several goodness-of-fit indices, such as the comparative fit index (CFI), the Tucker–Lewis index (TLI), and the root mean square error of approximation (RMSEA). Existing works have suggested that acceptable fit indices are evaluated by CFI and TLI values greater than 0.90 and a RMSEA less than 0.06 [70]. Lastly, this paper conducted bootstrapping analysis to test the significance of the indirect effect of CSR on counterproductive work behavior [70].

## 4. Results

### 4.1. Descriptive Statistics

Table 2 describes the results of the correlation analysis. The research variables, including CSR, organizational trust, organizational commitment, and counterproductive work behavior were significantly related to each other.

### 4.2. Measurement Model

This paper conducted CFA for all 30 items to test the discriminant validity of our five research variables (i.e., CSR, organizational trust, organizational commitment, counterproductive work behavior, and work overload) through the goodness-of-fit of the measurement model. This paper regarded a five-factor model as our hypothesized or baseline model. The result of our analysis showed that the hypothesized five-factor model has a good and acceptable fit (χ2 (df = 122) = 217.583; CFI = 0.977; TLI = 0.971; RMSEA= 0.048). Then, this study conducted a series of chi-square difference tests by consequently comparing the five-factor model to a four-factor model (χ2 (df = 126) = 853.972; CFI = 0.822; TLI = 0.784; RMSEA = 0.130), a three-factor model (χ2 (df = 129) = 1243.049; CFI = 0.728; TLI = 0.677; RMSEA = 0.159), a two-factor model (χ2 (df = 131) = 1641.395; CFI = 0.631; TLI = 0.569; RMSEA = 0.184), and a single-factor model (χ2 (df = 132) = 2190.111; CFI = 0.497; TLI = 0.417; RMSEA = 0.214) model. The results of the chi-square difference tests showed that the five-factor model had the best fit indices among the alternative models. Thus, the result indicates that the five variables have a proper degree of discriminant validity.

### 4.3. Structural Model

This study built a moderated mediation model that combines the sequential mediation structure with the moderation structure in the CSR–counterproductive work behavior link. The mediation structure led to CSR ⟶ organizational trust ⟶ organizational commitment ⟶ counterproductive work behavior, and the moderation structure suggests that work overload negatively moderates the association between CSR and organizational trust.

In addition, to create an interaction term between CSR and work overload, this study multiplied the two variables. Before multiplying, the two variables were centered on their means to reduce the multicollinearity. The centering method has been known to estimate the interaction term without influencing the correlations between the variables [71]. Then, to test whether multicollinearity bias exists between the independent variable (i.e., CSR) and moderator (i.e., work overload), this paper computed the value of variance inflation factors (VIFs) and tolerances [71]. The VIF values for CSR and work overload were 1.01 and 1.01, respectively. Moreover, the tolerance values were 0.99 and 0.99. However, the VIF scores were smaller than 10, with tolerance scores above 0.2; thus, this study proposes that CSR and work overload are relatively free from the issue of multicollinearity.

#### 4.3.1. Results of Mediation Analysis

**Figure 2:**
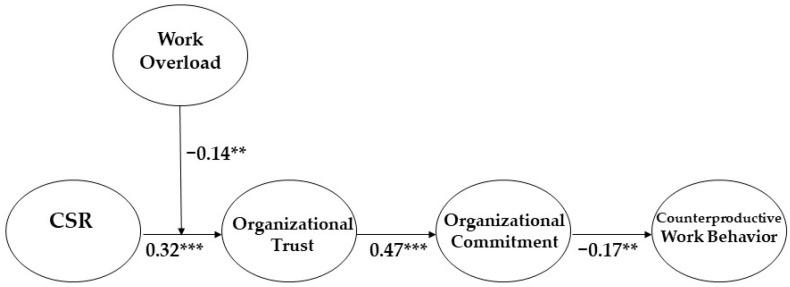
The final results of the research model. ** *p* < 0.01, *** *p* < 0.001.

To find the best mediation model, we compared a full mediation model to a partial mediation model by conducting a chi-square difference test with SEM analysis (see Figure 2). The partial mediation model has a direct path from CSR to counterproductive work behavior in addition to the full mediation model. The fit indices of all the models were good. The results of the chi-square difference test shows that the full mediation model (χ2 (df = 133) = 280.744; CFI = 0.952; TLI = 0.939; RMSEA = 0.057) has a better fit than the partial mediation model (χ2 (df = 132) = 280.743; CFI = 0.952; TLI = 0.938; RMSEA = 0.057), because the value of the chi-square difference between the models (i.e., 0.001) was statistically non-significant. The result indicates a possibility that CSR may indirectly influence the level of counterproductive work behavior.

The control variables (gender, position, tenure, and education level) were statistically non-significant. By including the control variables, the research model supported all hypotheses of this paper. CSR increases the level of organizational trust (β = 0.322, *p* < 0.001), supporting Hypothesis 1; the level of organizational trust increases the level of organizational commitment (β = 0.467, *p* < 0.001), supporting Hypothesis 2; and organizational trust diminishes the level of counterproductive work behavior (β = −0.172, *p* < 0.01), supporting Hypothesis 3 (Please see Figure 2). Based on the fact that the coefficient values of each path were highly significant, we can interpret that the strengths of the effects are adequate.

#### 4.3.2. Bootstrapping

This paper conducted bootstrapping analyses using a sample of 10,000 [70] to test Hypothesis 4, which is a sequential mediation hypothesis. The indirect mediation effect would be significant at the 5% level when the 95% bias-corrected confidence interval (CI) for the mean indirect mediation effect excludes zero [70]. The results demonstrated that the bias-corrected CI for the mean indirect effects on the paths did not include zero (95% CI = [−0.058, −0.006]). Thus, the result shows that Hypothesis 4 is supported. The direct, indirect, and total effects of the paths from CSR to counterproductive work behavior are shown in Table 3.

#### 4.3.3. Results of Moderation Analysis

**Figure 3:**
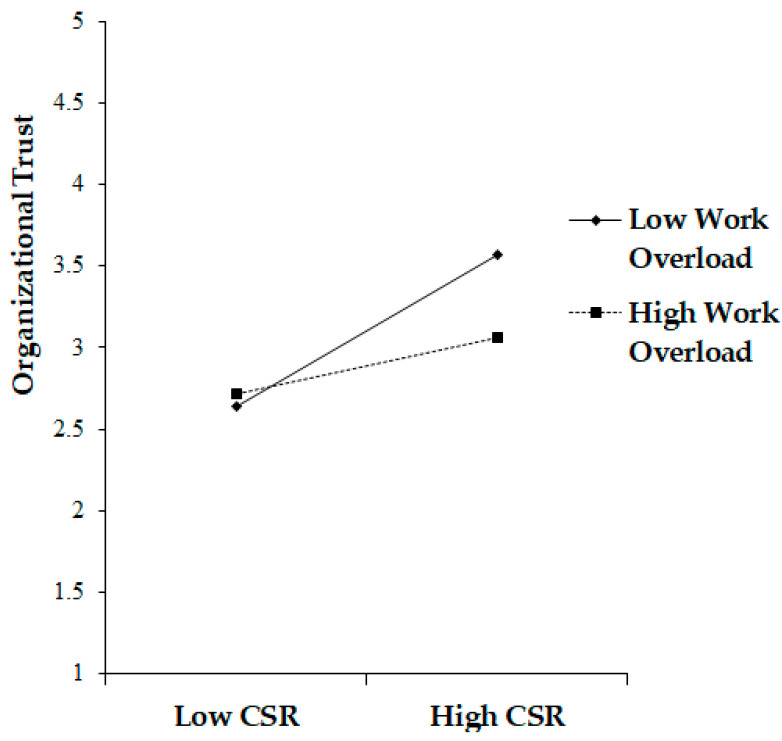
Moderating effect of work overload in the CSR–organizational trust link.

The moderating effect of work overload on the CSR–organizational trust link was tested by building a moderated mediation model. To create an interaction term, this paper performed a mean-centering process. When the coefficient of path from the interaction term to the organizational trust is significant, it indicates that work overload moderates the CSR–organizational trust link [70]. The coefficient value of the interaction term (β = −0.144, *p* < 0.01) showed that work overload negatively moderates the relationship, supporting Hypothesis 5 (Please See Figure 2). In other words, the relationship between CSR and organizational trust was weaker when the level of work overload was high than when it was low (Please See Figure 3).

## 5. Discussion

The purpose of the current paper was to investigate whether CSR decreases the level of counterproductive work behavior through sequentially enhancing the level of organizational trust and commitment. Furthermore, this paper tried to test the hypothesis that work overload may play a moderating role in the CSR–organizational trust link. To empirically test the hypotheses, this paper analyzed data gathered from 342 employees over three time points. The results of our analyses demonstrated that all the hypotheses were statistically supported. Theoretical and practical implications and limitations of the current paper are described as follows.

### 5.1. Theoretical Implications

We believe that the current research makes the following theoretical contributions. First, the current study investigated the micro-foundation of corporate social responsibility. The many previous works on CSR tend to concentrate mainly on the association between CSR and external stakeholders (e.g., customers, local communities, suppliers, and the environment), investigating the influence of CSR on macro-level outcomes (e.g., financial performance, reputation of firm, and loyalty of consumers). However, the primary players who substantially plans and implements the social responsibility practices in an organization are its members. They also translate the firm’s socially responsible activities into the various organizational outcomes, including at both the macro- and micro-levels [2,9,10]. From this point of view, the extant works on CSR tend to pay insufficient attention to the reactions of employees toward the CSR. This research may make a theoretical contribution to the CSR literature by taking the micro-level perspective.

Second, this paper examined the impact of CSR on employee’s negative behavior, i.e., counterproductive work behavior. Although the previous works have mainly focused on the impacts of CSR on the positive perceptions or attitudes, including organizational commitment, work engagement, job satisfaction, meaningfulness of work, and in-/extra-role performance, those studies paid relatively less attention to CSR’s influences on employees’ negative behavior. Although perceptions or attitudes are critical factors in an organization, these are likely to be ultimately expressed in the form of “behavior” in a real organizational context. Thus, employee’s behaviors are likely to be more directly related to macro-level organizational outcomes, including financial performance, compared to the employee’s perceptions or attitudes. In addition, the research examining the impact of CSR on an employee’s behavior tends to primarily deal with “positive” behaviors, including organizational citizenship behavior or innovative behavior, and not negative behaviors. Given that both positive and negative aspects exist in a real organization, and that positive and negative aspects of a member’s behavior have different processes or dimensions in an organization [9,10], investigating the influence of CSR activities on counterproductive work behavior may contribute to improving the corporate social responsibility and counterproductive work behavior literature.

Third, the current study investigated the intermediating mechanism of the influence of CSR activities on counterproductive work behavior through providing mediators and moderator in the relationship. Based on a context–attitude–behavior framework [29], the influential model of trust [34], and social exchange theory [42], this study shows that CSR decreases the level of counterproductive work behavior by sequentially enhancing the level of members’ organizational trust and commitment. This result means that the achievement of the spirit of CSR would positively contribute to decreasing the negative outcomes in an organization by enhancing employee’s perception or attitudes. By explaining the reason why corporate social responsibility can decrease an employee’s negative behavior, the current paper may contribute to connecting the CSR literature with the counterproductive work behavior literature.

Fourth, the current research empirically showed that employee’s work overload plays an important contingent role in the CSR–organizational trust link. In other words, the positive influence of CSR on an employee’s organizational trust does not appear to all employees in the same way, depending on the degree of each employee’s work overload. For instance, for a member with a high degree of work overload, no matter how actively a firm implements CSR activities, the employee’s organizational trust may not be increased enough. On the contrary, an employee with a low level of work overload may positively respond to the firm’s CSR, boosting the positive influence of CSR on organizational trust. The current research may positively contribute to both the CSR and work overload literature by examining the contingent factor that negatively moderates the positive influence of CSR on the organizational trust link.

Fifth, the current study sheds light on the critical role of “authenticity”, which is reflected in the degree of an employee’s work overload. To support this, this paper empirically demonstrated that the employee’s work overload negatively moderates the CSR–organizational trust link. This result means that members do not simply trust in their company’s good will (i.e., CSR) without doubt if their workload is excessive. Regardless of how proactively the organization implements CSR practices, the positive impact of CSR on members’ attitudes (e.g., organizational trust and commitment) is decreased if the level of an employee’s work overload is high, because it makes them think that the CSR is not authentic. The important aspect for the good activity (i.e., CSR) is whether it is implemented with authenticity.

### 5.2. Practical Implications

We believe that this research may provide some practical implication for top management teams and leaders in an organization. First, this paper suggests that top management teams and leaders are required to understand that the moral acts (i.e., CSR) are not just “costs” to perform social “obligations” [9,10]. If a firm satisfies its social responsibilities, the quality of employees’ perceptions, attitudes, and behaviors will be enhanced. Specific, through the CSR practices of the firm, the employees may undergo an increased level of organizational trust and commitment, thus diminishing the degree of negative behaviors such as counterproductive work behavior. The CSR activities can be an active “investment” to facilitate positive organizational outcomes [2,9,10].

Second, the results of the current research may provide top management teams and leaders with the practical insight that they should monitor members’ degree of organizational trust and commitment to check if the CSR practices positively influence member’s perceptions, attitudes, and behaviors. The results demonstrated that CSR diminishes the employees’ counterproductive behavior via sequentially facilitating the degree of organizational trust and commitment. This indicates that the degree of organizational trust and commitment may play a role as criteria to check whether the positive impacts of CSR activities are working in the organization. In other words, if the levels of organizational trust and commitment are not increased despite active implementation of the CSR practices, the CSR may not effectively work in the organization.

Third, this paper may help corporate leaders to understand that all members of the organization do not always respond to the CSR activities in the same way. In other words, there exist individual differences of the members in an organization. Regardless of how well a firm performs its social responsibilities, if an employee of the firm feels a considerable level of job instability, they will not experience enough psychological safety through the moral behaviors of the company. In contrast, when an employee perceives a low level of job insecurity, the positive effects of CSR activities on the employee’s psychological safety may be amplified due to their positive response to the moral practices.

### 5.3. Limitations and Suggestions for Future Studies

*Although we believe that this research* makes some theoretical and practical contributions, as described above, there are some limitations that should be addressed. First, the current study could not evaluate the level of CSR practices in an objective way since the study depended on survey data from respondents’ self-reporting. The level of objective CSR, such as the amount of investment undertaken to implement various kinds of CSR activities, may not directly influence employees’ perceptions and attitudes, because the objective investment should be interpreted by the employees’ sense-making process. Nonetheless, the objective data also may be reflected in employees’ reactions toward CSR activities. Thus, future studies are required to compare the differential impacts of the objective CSR and the perceived CSR on employee’s perceptions, attitudes, and behaviors.

Second, although the essential values of CSR practices are common in Western and Eastern societies [72,73], it is possible that there exist cultural differences in terms of members’ attitudes or perceptions towards CSR. Given that the South Korean economy has rapidly grown, members of Korean organizations are likely to be relatively less reactive to these kinds of ethical practices, such as CSR, compared to the members of Western firms [72,74]. The current research could not adequately deal with this issue because this study only used data from Korean firms. Thus, the results of this paper should be cautiously interpreted in different cultures [72,75].

Third, it was not possible to ensure that the main research constructs of this study (i.e., CSR, work overload, organizational trust, organizational commitment, and counterproductive work behavior) were stable during the entire timespan (i.e., three time points) for a particular participant because the research design was only time-lagged, not longitudinal. Thus, there may be a possibility that the main research variables were changed between Times 1, 2, and 2. For example, this paper measured the level of CSR at its initial level, and later measured its consequences (i.e., organizational trust, commitment) when the level of CSR was lower. We suggest that future research should re-test the hypotheses by utilizing strict longitudinal data.

Fourth, this paper could not adequately deal with the issue of sampling bias because the data-collection processes were conducted via smartphone- and Internet-based methods. As a result, the group of elderly people who are not familiar with the updated techniques may be under-represented. This can be a critical cause of sampling bias. Future studies should consider this issue through various methods to decrease the bias.

## 6. Conclusions

Based on a context–attitude–behavior framework, this paper examined the impact of CSR on counterproductive work behavior. The results showed that CSR diminished the degree of members’ counterproductive work behavior through sequentially increasing the degree of organizational trust and commitment. In addition, work overload played a role of moderator in the CSR–organizational trust link. This means that members’ organizational trust and commitment are an underlying mechanism in translating CSR into their negative behavior (i.e., counterproductive work behavior). Furthermore, their work overload negatively moderated the positive influence of CSR on their behavior. Although the current paper has some limitations, we believe that this research may contribute to improving CSR and counterproductive work behavior literature by demonstrating the intermediating mechanism and its contingent factor in the link.

## Figures and Tables

**Table 1:** Descriptive features of the sample.

Characteristic	Percent
**Gender**	
Male	50.3%
Female	49.7%
**Age (years)**	
20–29	14.6%
30–39	36.3%
40–49	34.2%
50–59	4.9%
**Education**	
Below high school	8.5%
Community college	18.4%
Bachelor’s degree	60.8%
Graduate degree	12.3%
**Position**	
Staff	24.0%
Assistant manager	22.8%
Manager	23.4%
Deputy general manager	10.2%
Department/general manager and above director	19.6%
**Tenure (years)**	
**Under 5**	48.0%
6 to 10	26.9%
11 to 15	12.2%
16 to 20	7.2%
21 to 25	2.3%
Above 25	3.4%
**Industry type**	
Manufacturing	24.9%
Services	19.3%
Construction	12.1%
Information service and telecommunications	9.1%
Education	7.3%
Health and welfare	10.5%
Financial/insurance	3.2%
Others	13.6%
Manufacturing	24.9%
Services	19.3%
Construction	12.1%

**Table 2:** Descriptive statistics.

	1	2	3	4	5	6	7	8
1. Gender_T2	-							
2. Education	−0.10	-						
3. Tenure_T2	−0.27 **	0.004	-					
4. Position_T2	−0.41 **	0.26 **	0.27 **	-				
5. CSR_T1	−0.21 **	0.12 *	0.19 **	−0.14 *	-			
6. Work Overload_T1	−0.17 **	−0.02	0.10	0.04	0.09	-		
7. OT_T2	−0.09	0.14 *	0.11 *	0.16 **	0.37 **	−0.09	-	
8. OC_T2	−0.22 **	−0.14 *	0.26 **	0.23 **	0.45 **	−0.06	0.51 **	-
9. CWB_T3	−0.09	−0.06	0.02	−0.01	−0.05	0.31 **	−0.26 **	−0.15 **

Note: * *p* < 0.05. ** *p* < 0.01. OT means organizational trust, OC means organizational commitment, and CWB means counterproductive work behavior.

**Table 3:** Direct, indirect, and total effects of the final research model.

Model	Direct Effects	Indirect Effects	Total Effects
CSR -> organizational trust -> organizational commitment -> counterproductive work behavior	0.000	−0.026	−0.026

All values are standardized.

## Data Availability

No new data were created or analyzed in this study. Data sharing is not applicable to this article.

## Appendix A. Measures

**Table A1:** The Items and the Value of Cronbach Alpha of Each Variable.

Variable	Items	Cronbach Alpha
CSR	1. “Our company participates in activities which aim to protect and improve the quality of the natural environment”.	0.91
	2. “Our company implements special programs to minimize its negative impact on the natural environment”.	
	3. “Our company targets sustainable growth which considers future generations”.	
	4. “Our company contributes to campaigns and projects that promote the well-being of society”.	
	5. “Our company emphasizes the importance of its social responsibilities to society”.	
	6. “Our company actively participates in voluntarily donations to charities and nongovernmental organizations”.	
	7. “Management at our company is primarily concerned with employees’ needs and wants”.	
	8. “Our company policies encourage employees to develop their skills and careers”.	
	9. “Our company supports employees’ growth and development”.	
	10. “Our company respects consumer rights beyond legal requirements”.	
	11. “Our company provides full and accurate information about its products to its customers”.	
	12. “Customer satisfaction is highly important for our company”.	
Work Overload	1. “I am pressured to work long hours”.	0.91
	2. “I have unachievable deadlines”.	
	3. “I have to work very fast”.	
	4. “I have to work very intensively”.	
	5. “I have unrealistic time pressures”.	
Organizational Trust	1. “I trust my organization”.	0.90
	2. “I feel that my company is reliable”.	
	3. “My organization is trustworthy and reliable”.	
Organizational Commitment	1.“I really feel as if my organization’s problems are my own”.	0.93
	2. “I feel a strong sense of belonging to my organization”.	
	3. “I feel emotionally attached to my organization”.	
	4. “I would be very happy to spend the rest of my career working with my organization”.	
	5. “I feel like part of the family at my organization.”	
Counterproductive Work Behavior	1. “This employee told people outside the job what a lousy place you work for”.	0.89
	2. “This employee insulted someone about their job performance”.	
	3. “This employee purposely worked slowly when things needed to get done”.	
	4. “This employee complained about insignificant things at work”.	
	5. “This employee started an argument with someone at work”.	
